# Supplementary material for: Comparison of the Effectiveness of Lifestyle Modification with Other Treatments on the Incidence of Type 2 Diabetes in People at High Risk: A Network Meta-Analysis
Source: Nutrients. 2019 Jun 19;11(6):1373. doi: 10.3390/nu11061373 (PMC6627198; doi:10.3390/nu11061373)
Supplement: Supplementary file 1 [file nutrients-11-01373-s001.zip › nutrients-507499-tableS1.pdf]

Table S1. Inclusion criteria for the meta-analysis (PICO)

PICO	Description
Population	Patients who were at high risk of type 2 diabetes. (Defined as IGT or IFG. In addition to IGT or IFG, patients with overweight or obesity were included).
Intervention	Lifestyle modification (combined diet and exercise) [L], dietary intervention alone [D], exercise intervention [E], Orlistat [O], metformin (or flumamine) [M], acarbose and voglibose [A], pioglitazon and rosiglitazone [T], pitavastatin [PI], glipizide [G,SU], herbal medicines such as the Jiangtang bushen recipe or Tianqi [H].<br>Mediterranean diet was treated as dietary interventions but in the additional analysis it was also conducted as an independent intervention.
Comparison	Standard nutritional recommendations [S] or no treatment including placebo [P]
Outcome	Number of type 2 diabetes at 1-year follow-up
