# Supplementary material for: Comparison of the Effectiveness of Lifestyle Modification with Other Treatments on the Incidence of Type 2 Diabetes in People at High Risk: A Network Meta-Analysis
Source: Nutrients. 2019 Jun 19;11(6):1373. doi: 10.3390/nu11061373 (PMC6627198; doi:10.3390/nu11061373)
Supplement: Supplementary file 1 [file nutrients-11-01373-s001.zip › nutrients-507499-tableS2.pdf]

Table S2. Summary table

NO	Trial	year	REF #	Country/Area	age	BMI (kg/m^2)	follow-up year	study full follow-up year	Control			Intervention									subject type	other (study design, subgroup, special data calcuration)
									treat ment	r_c	n_c	treat ment	r_i	n_i	treat ment	r_i	n_j	treat ment	r_i	n_j		
1	Pan	1997	21	Da Qing, China	45	26	6		S	90	133	D	57	130	E	58	141	L	58	126	IGT	cluster RCT
2	Fracog	1999	22	Australia	39	25	4.25[Q2]		S	27	96	D	26	97							IGT	
3	Li	1999	23	China	30	26	1		P	6	43	M	3	42							IGT	
4	Heymsfield	2000	24	USA&Europe	44	36	2		P	4	53	O	2	67							IGT obesity	subgroup
5	Lindstrom	2003	25	Finland, DPS	55	31	3[M]		S	51	250	L	22	256							IGT overweight	
6	Oldroyd	2006	34	UK	58	30	2		S	8	32	L	7	37							IGT	
7	Chiasson	2002	26	STOP-NIDDM, International multi-center	55	31	3.3[M]		P	285	686	A	221	682							IGT overweight	
8	Knowler	2002	27	USA, DPPRG	51	34	1	2.8[M]	S	119	1082	L	52	1079	M	84	1073				IGT	from Fig. 2
9	Liao	2002	28	USA	54	26	1	2	S	2	32	L	1	32							IGT	
10	Watanabe	2003	29	Japan	55	24	1		S	6	87	L	3	86							IGT	
11	Tao	2004	30	China	51	25	3		S	13	32	E	4	28							IGT	
12	Fan	2005	31	China	56	26	1		P	3	26	H	1	25							IGT	
13	Torgerson	2004	32	Sweden, XENDOS	44	37	1	4	P	28	344	O	14	350							IGT	subgroup, from Fig.1
14	Kosaka	2005	33	Japan	52	24	4		S	33	356	L	3	102							IGT	
15	DREAM	2006	35	International multi-center	55	31	3[Q2]		P	489	2646	R	449	2623							IGT/IFG	2x2 design
16	Eriksson	2006	36	Finland, Marmo	57	28	1.5		P	5	17	G	1	17							IGT	
17	Ramachandran	2006	37	India, IDDP	46	26	3		S	73	133	L	47	120	M	52	128				IGT	
18	Roumen	2008	38	SLIM	56	29	1	3	S	9	47	L	0.5	44							IGT	
19	Kawahara	2008	39	Japan (DES)	51	25	3		S	19	142	L	15	141							IGT	
20	Kawamori	2009	40	Japan	56	26	1	3	P	83	881	A	32	897							IGT	
21	Penn	2009	41	UK, EDIPS	57	34	1	3[M]	S	3	40	L	3	43							IGT	
22	Ramachandran	2009	42	India, IDDP-2	45	26	3		P	64	203	T	61	204							IGT	from Fig.2
23	Yates	2009	43	UK	65	29	1		S	6	34	L	2	31	E	0	33				IGT overweight	from Fig.
24	Zinman	2010	44	Canada	55	32	3.9[Q2]		P	41	104	M	14	103							IGT overweight	
25	DeFronzo	2011	45	USA	53	34	2.4[Q2]		P	50	299	T	15	303							IGT	
26	Gagnon	2011	46	Canada	57	35	1		S	3	26	L	0.5	22							IFG/IGT overweight	
27	Lu	2011	47	China	63	27	2		S	5	41	M	0.5	46							IFGorIGT	2x2 design
28	Lu	2011	47	China	63	27	2		S	3	45	A	0.5	49							IGT	
29	Salas-Salvadó	2011	48	Spain , PREDIMED-Reus	67	30	1	4[Q2]	S	2	134	D	1	284							IGT	Intervention= VOO+NUTS
30	Sakane	2011	49	Japan, JDPP	51	25	1	3	S	7	131	L	1	123							IGT	
31	Zhou	2011	50	China	63	24	0.5		S	5	58	E	0.5	58	D	0.5	57	L	0.5	59	IGT	cluster RCT
32	Saito	2011	51	Japan, FINT	49	27	2.7[M]		S	41	131	L	23	131							IGT/IFC overweight	subgroup
33	Ockene	2012	52	Latino, USA	52	34	1	2.5	S	9	150	L	9	162							IGT	
34	Vermunt	2012	53	Dutch	51	25	1.5	2.5	S	32	386	L	33	407							IGT	from percentages
35	Odawara	2013	54	Japan, JPREDIC	-	-	5	5.5	L	99	534	T	91	556							IGT	from Cumurative Inidence
36	Wong	2013	55	China	55	26	1	2	S	8	50	L	3	54							IGT/IFG	
37	Xu	2012	56	China	58	26	1		S	7	40	L	6	41							IGT	
38	Fang	2014	57	China	55	25	1		P	33	216	H	19	223							IGT	
39	Lian	2014	58	China	53	25	1		P	56	191	H	36	198							IGT	
40	Weber	2016	59	India, DCLIP	44	28	1	3	S	14	281	M	5	269							IGT/IFG	
	Total subjects= 20111										10079			####								

S: standard, P: Placebo, L: lifestyle modification, E: exercise, D: Diet, A: Acarbose/Voglibose, M: Metformin/Flumamine, T(diabetes drugs): Glipizide, Pioglitazon, Rosiglitazone, P: Pitavastatin, O: Orlistat (anti-obesity drug), H: Herbal medicine/ Juangtang bushen recipe/ Tianqi

Q2: Median, M: Mean
