# Supplementary material for: Comparison of the Effectiveness of Lifestyle Modification with Other Treatments on the Incidence of Type 2 Diabetes in People at High Risk: A Network Meta-Analysis
Source: Nutrients. 2019 Jun 19;11(6):1373. doi: 10.3390/nu11061373 (PMC6627198; doi:10.3390/nu11061373)
Supplement: Supplementary file 1 [file nutrients-11-01373-s001.zip › nutrients-507499-tableS3.pdf]

Table S3. Search Code

Therapy/Broad[filter] AND (high[All Fields] AND ("risk"[MeSH Terms] OR "risk"[All Fields]) AND ("diabetes mellitus, type 2"[MeSH Terms] OR "type 2 diabetes mellitus"[All Fields] OR "diabetes type 2"[All Fields]) AND ("life style"[MeSH Terms] OR ("life"[All Fields] AND "style"[All Fields]) OR "life style"[All Fields] OR "lifestyle"[All Fields] AND ("methods"[MeSH Terms] OR "methods"[All Fields] OR "intervention"[All Fields]) AND ("random allocation"[MeSH Terms] OR ("random"[All Fields] AND "allocation"[All Fields]) OR "random allocation"[All Fields] OR "randomized"[All Fields]) AND ("clinical trials as topic"[MeSH Terms] OR ("clinical"[All Fields] AND "trials"[All Fields] AND "topic"[All Fields]) OR "clinical trials as topic"[All Fields] OR "trial"[All Fields]) AND ("adult"[MeSH Terms] OR "adult"[All Fields] OR "adults"[All Fields]))

N=222 (2018/11/29) **Search results** **Items: 1 to 200 of 222**
