# Supplementary material for: Comparison of the Effectiveness of Lifestyle Modification with Other Treatments on the Incidence of Type 2 Diabetes in People at High Risk: A Network Meta-Analysis
Source: Nutrients. 2019 Jun 19;11(6):1373. doi: 10.3390/nu11061373 (PMC6627198; doi:10.3390/nu11061373)
Supplement: Supplementary file 1 [file nutrients-11-01373-s001.zip › nutrients-507499-tableS4.pdf]

Table S4. NMA for separating Mediterranean diet from Diet intervention using 1yr and full follow-up study duration with BMI groups (frequentist approach, random-effect model)

	1Yr with Mediterranean	Full yr with Mediterranean	Full yr with Mediterranean (BMI<30)	Full yr with Mediterranean (BMI>=30)
	OR [95% CI]	OR [95% CI]	OR [95% CI]	OR [95% CI]
**Comparison for Lifestyle**				
Lifestyle vs. Exercise (indirect)	1.18 [0.74, 1.93]	1.13 [0.75, 1.83]	1.10 [0.87, 1.40]	-
Lifestyle vs. Diet (indirect)	0.90 [0.59, 1.40]	0.88 [0.60, 1.28]	0.64 [0.56, 0.73]	-
Lifestyle vs. Mediterranean diet	2.56 [0.22, 30.3]	1.06 [0.54, 2.12]	-	0.96 [0.32, 2.91]
Lifestylevs. Orlistat (indirect)	1.19 [0.55, 2.58]	0.91 [0.54, 1.53]	-	0.82 [0.38, 1.79]
Lifestyle vs. Acarbose/Voglibose (indirect)	0.92 [0.57, 1.48]	0.84 [0.56, 1.25]	1.27 [0.36, 4.42]	0.68 [0.25, 1.83]
Lifestyle vs. Metformin/Flumamine (indirect)	0.86 [0.60, 1.25]	0.73 [0.60, 1.05]	0.89 [0.73, 1.10]	0.73 [0.34, 1.56]
Lifestyle vs. Pioglitazon, Rosiglitazone (indirect)	0.57 [0.32, 1.00]	0.57 [0.36, 1.29]	0.61 [0.17, 2.15]	0.68 [0.26, 1.78]
Lifestyle vs. Pitavastatin (indirect)	0.68 [0.36, 1.29]	0.72 [0.42, 1.24]	0.79 [0.59, 1.05]	-
Lifestyle vs. Glipizide (indirect)	2.07 [0.24, 17.86]	2.16 [0.26, 17.86]	2.90 [0.27, 31.25]	-
Lifestyle vs. Herbal medicine (indirect)	0.71 [0.38, 1.36]	0.74 [0.43, 1.28]	0.98 [0.28, 3.44]	-
Lifestyle vs. Standard	0.60 [0.48, 0.76]	0.59 [0.49, 0.70]	0.64 [0.56, 0.73]	0.53 [0.30, 0.94]
Lifestyle vs. Placebo	0.41 [0.27, 0.63]	0.43 [0.31, 0.61]	0.58 [0.17, 1.97]	0.40 [0.19, 0.82]
**Comparison with Standard**				
Diet vs. Standard	0.67 [0.44, 1.01]	0.67 [0.46, 0.95]	0.68 [0.55, 0.83]	-
Mediterranean diet vs. Standard	0.24 [0.02, 2.73]	0.55 [0.28, 1.07]		0.55 [0.21, 1.42]
Exercise vs. Standard	0.51 [0.32, 0.81]	0.52 [0.34, 0.78]	0.58 [0.47, 0.72]	-
Orlistat vs. Standard	0.51 [0.24, 1.07]	0.64 [0.39, 1.05]	-	0.64 [0.36, 1.14]
Acarbose/Voglibose vs. Standard	0.66 [0.42, 1.04]	0.70 [0.48, 1.05]	0.50 [0.15, 1.75]	0.78 [0.35, 1.76]
Metformin/Flumamine vs. Standard	0.70[0.48, 1.03]	0.74 [0.56, 0.98]	0.72 [0.60, 0.86]	0.72 [0.29, 1.80]
Pioglitazon, Rosiglitazone vs. Standard (indirect)	0.66 [0.42, 1.04]	1.02 [0.64, 1.62]	1.05 [0.30, 3.66]	0.77 [0.26, 2.27]
Pitavastatin vs. Standard	0.88 [0.49, 1.59]	0.81 [0.49, 1.35]	0.81 [0.63, 1.05]	-
Glipizide vs. Standard (indirect)	0.29 [0.03, 2.51]	0.27 [0.03, 2.24]	0.22 [0.02, 2.37]	-
Herbal medicine vs. Standard (indirect)	0.85 [0.44, 1.61]	0.79 [0.46, 1.37]	0.66 [0.19, 2.29]	-
Placebo vs. Standard (indirect)	1.46 [0.95, 2.25]	1.36 [0.96, 1.91]	1.10 [0.33, 3.73]	1.33 [0.55, 3.21]
**Comparison with Placebo**				
Diet vs. Placebo (indirect)	0.46 [0.26, 0.82]	0.49 [0.30, 0.80]	0.62 [0.18, 2.11]	-
Mediterranean diet vs. Placebo (indirect)	0.16 [0.01, 1.94]	0.41 [0.19, 0.86]	-	0.41 [0.11, 1.50]
Exercise vs. Placebo (indirect)	0.35[0.19, 0.64]	0.38[0.23, 0.65]	0.53[0.15, 1.81]	-
Orlistat vs. Placebo	0.35 [0.15, 0.79]	0.48 [0.27, 0.85]	-	0.48 [0.18, 1.28]
Acarbose/Voglibose vs. Placebo	0.45 [0.28, 0.74]	0.52 [0.34, 0.77]	0.46 [0.33, 0.63]	0.59 [0.18, 1.94]
Metformin/Flumamine vs Placebo	0.48 [0.32, 0.71]	0.55 [0.39, 0.76]	0.65 [0.19, 2.17]	0.54 [0.29, 1.01]
Pioglitazon, Rosiglitazone vs. Placebo	0.73 [0.50, 1.05]	0.75 [0.55, 1.04]	0.95 [0.71, 1.27]	0.58 [0.31, 1.10]
Pitavastatin vs. Placebo (indirect)	0.60 [0.29, 1.25]	0.60 [0.33, 1.10]	0.73 [0.21, 2.55]	-
Glipizide vs. Placebo	0.20 [0.02, 1.64]	0.20 [0.02, 1.61]	0.20 [0.03, 1.54]	-
Herbal medicine (Juangtang bushen recipe, Tianqi) vs. Placebo	0.58 [0.34, 0.93]	0.58 [0.38, 0.90]	0.59 [0.44, 0.80]	-
Log Likeyhood Ratio/p-values for inconsistency	X^2=7.74, p=0.74 (df= 11	X^2=7.70, p=0.74 (df= 11	X^2=9.06, p=0.43 (df= 9	X^2=1.58, p=0.45 (df= 2)

For NMA with random effeccts model

Herbal medicine includes Juangtang bushen recipe, and Tianqi.
