# Supplementary material for: Comparison of the Effectiveness of Lifestyle Modification with Other Treatments on the Incidence of Type 2 Diabetes in People at High Risk: A Network Meta-Analysis
Source: Nutrients. 2019 Jun 19;11(6):1373. doi: 10.3390/nu11061373 (PMC6627198; doi:10.3390/nu11061373)
Supplement: Supplementary file 1 [file nutrients-11-01373-s001.zip › nutrients-507499-figure S1.pdf]

	Published year	Refference number	Random sequence generation	Allocation concealment	Blinding of participants and personn	Blinding of outcome assessment	Incomplete outcome data	Selective reporting	Overall Judgement
Pan、Da Qing	1997	21	?	?	-	+	?	?	H
Fracog	1999	22	?	+	-	+	+	?	L
Li	1999	23	?	+	+	+	-	+	M
Heymsfield	2000	24	?	-	-	+	-	-	H
Lindström, DPS	2003	25	+	+	-	+	+	+	L
Oldroyd	2006	34	+	+	-	?	+	?	M
Chiasson, STOP-NIDDM	2002	26	+	+	+	+	+	+	L
Knower, DPP	2002	27	?	+	-	+	+	+	L
Liao	2002	28	+	+	-	+	+	?	L
Watanabe	2003	29	+	+	-	+	+	+	M
Tao	2004	30	-	-	-	?	?	?	H
Fan	2005	31	+	?	?	?	+	?	H
Torgerson, XENDOS	2004	32	?	+	?	+	+	+	M
Kosaka	2005	33	?	-	-	+	+	?	H
DREAM	2006	35	+	+	+	+	+	+	L
Eriksson, MARMO	2006	36	?	-	+	+	+	?	M
Ramachandr, IDPP	2006	37	?	+	-	+	+	?	L
Roumen, SLIM	2008	38	+	+	-	+	+	+	M
Kawahara	2008	39	+	+	-	+	?	+	L
Kawamori	2009	40	+	+	+	+	+	+	M
Penn, EDIPS	2009	41	+	+	+	+	+	+	L
Ramachandran, IDPP	2009	42	+	+	+	+	+	+	M
Yates	2009	43	+	+	-	+	+	?	M
Zinman	2010	44	+	+	+	+	+	+	L
DeFronzo	2011	45	+	+	+	+	+	+	L
Gagnon	2011	46	+	?	-	+	+	?	M
Lu	2011	47	?	?	-	+	+	?	H
Lu	2011	47	?	?	-	+	+	?	H
Salas-Salvado, PREDMED	2011	48	?	?	-	+	?	?	L
Sakane	2011	49	+	?	-	+	?	?	M
Zhou	2011	50	?	?	-	+	?	?	H
Saito, FINT	2011	51	+	+	-	+	?	?	H
Oskene	2012	52	?	?	-	+	+	+	M
Vermunt	2012	53	?	?	-	+	+	?	H
Odawara, JDPP?	2013	54	?	+	+	+	+	+	M
Wong	2013	55	+	+	-	+	+	?	L
Xu	2012	56	+	?	-	+	?	?	H
Fang	2014	57	?	?	-	+	?	?	H
Lian	2014	58	+	+	-	+	+	+	M
Weber, DCLIP	2016	59	+	+	-	+	+	+	M

+' Low risk of bias, '-' High risk of bias, '?' Unclear
