# Supplementary material for: Comparison of the Effectiveness of Lifestyle Modification with Other Treatments on the Incidence of Type 2 Diabetes in People at High Risk: A Network Meta-Analysis
Source: Nutrients. 2019 Jun 19;11(6):1373. doi: 10.3390/nu11061373 (PMC6627198; doi:10.3390/nu11061373)

Figure S2. Funnel plots

a) Funnel plot for lifestyle modification effects on the onset of type 2 diabetes.

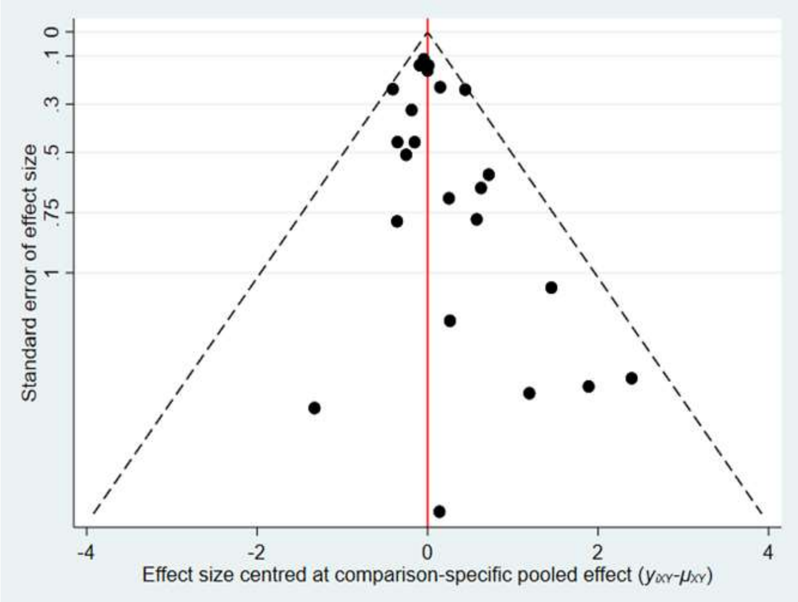

b) Filled funnel plot with pseudo 95% confidence limits using trim and fill method for lifestyle modification effects on the onset of type 2 diabetes.

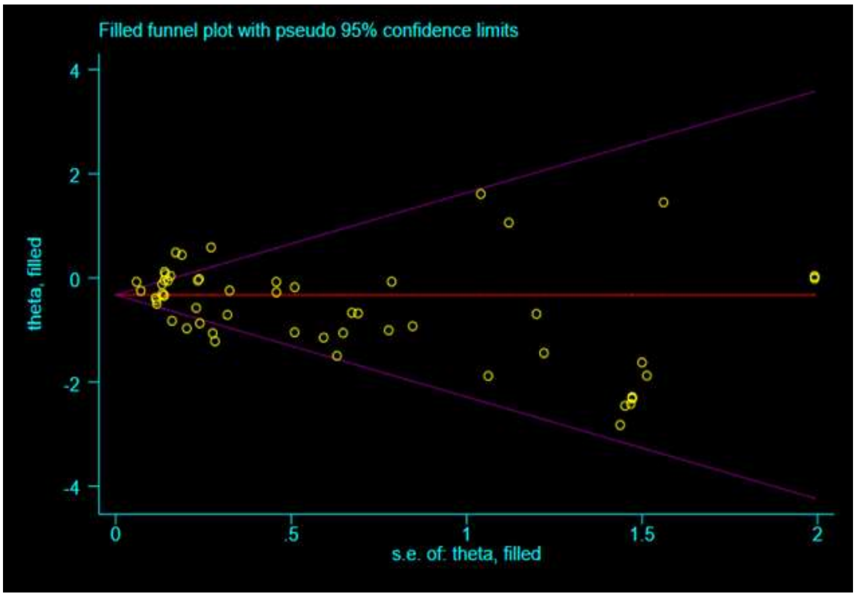

Supplement: Supplementary file 1 [file nutrients-11-01373-s001.zip › nutrients-507499-figure S2.pdf]
